# A systems-based partnership learning model for strengthening primary healthcare

**DOI:** 10.1186/1748-5908-8-143

**Published:** 2013-12-17

**Authors:** Ross Bailie, Veronica Matthews, Jenny Brands, Gill Schierhout

**Affiliations:** 1 Menzies School of Health Research, Charles Darwin University, 1/147 Wharf Street, Brisbane, Spring Hill, Australia

**Keywords:** Health systems strengthening, Quality improvement, Comprehensive primary healthcare, Participatory, Partnership, Learning, Information

## Abstract

**Background:**

Strengthening primary healthcare systems is vital to improving health outcomes and reducing inequity. However, there are few tools and models available in published literature showing how primary care system strengthening can be achieved on a large scale. Challenges to strengthening primary healthcare (PHC) systems include the dispersion, diversity and relative independence of primary care providers; the scope and complexity of PHC; limited infrastructure available to support population health approaches; and the generally poor and fragmented state of PHC information systems.

Drawing on concepts of comprehensive PHC, integrated quality improvement (IQI) methods, system-based research networks, and system-based participatory action research, we describe a learning model for strengthening PHC that addresses these challenges. We describe the evolution of this model within the Australian Aboriginal and Torres Strait Islander primary healthcare context, successes and challenges in its application, and key issues for further research.

**Discussion:**

IQI approaches combined with system-based participatory action research and system-based research networks offer potential to support program implementation and ongoing learning across a wide scope of primary healthcare practice and on a large scale. The Partnership Learning Model (PLM) can be seen as an integrated model for large-scale knowledge translation across the scope of priority aspects of PHC. With appropriate engagement of relevant stakeholders, the model may be applicable to a wide range of settings. In IQI, and in the PLM specifically, there is a clear role for research in contributing to refining and evaluating existing tools and processes, and in developing and trialling innovations. Achieving an appropriate balance between funding IQI activity as part of routine service delivery and funding IQI related research will be vital to developing and sustaining this type of PLM.

**Summary:**

This paper draws together several different previously described concepts and extends the understanding of how PHC systems can be strengthened through systematic and partnership-based approaches. We describe a model developed from these concepts and its application in the Australian Indigenous primary healthcare context, and raise questions about sustainability and wider relevance of the model.

## Background

Health systems around the world are struggling to respond to multiple challenges in a complex and constantly changing world. Profound levels of inequity in health status continue to exist globally and within nations. The World Health Organization (WHO) [1] has shifted its focus to the strengthening of health systems and has proposed systems thinking as a way to build capacity to meet these challenges. Systems thinking encourages the dynamic engagement of diverse stakeholders and aims to inspire system-wide learning, planning, evaluation and research [2].

The delivery of effective, good quality care is the first of the WHO’s widely adopted ‘six building blocks’ for health system strengthening [1]. Quality improvement (QI) is one mechanism for enhancing the overall quality of care provided within a health system [3,4]. Systematic review evidence suggests that system-wide QI approaches are associated with the largest effects [5], and that while no one model of QI is more effective than others, the conditions for effective QI include the use of multi-faceted approaches that are well integrated with local context, involve sustained action and engagement across multiple levels, address systemic barriers through interventions across multiple levels, IT systems that provide timely data for measuring performance, and well-trained staff [6]. The core of QI is ongoing learning and improvement, and this makes it suited to systems thinking approaches. Such systematic learning processes, along with research and evaluation, are identified as important contributors to a robust adaptive health system [6]. However, the quality improvement literature appears to have paid little attention to systematic research or evaluation. Even in the best described QI models there have been barriers to the sharing of data within and across organizations for improvement, learning, or research purposes, and limited evidence of better patient or clinical outcomes [6].

This paper describes a Partnership Learning model (PLM) that uses an integrated QI (IQI) approach in primary healthcare, with an emphasis on systematically generating and using evidence for health system strengthening. The paper also lays the foundation for separate papers that demonstrate how application of the model has enabled generation of primary healthcare system performance data that have previously not been available and provide empirical evidence of improvements in quality of care. The focus of the model is on comprehensive (or community-based) primary healthcare as arguably the most important part of the health system in any country [7,8]. We provide a general description of the key components of the model and how these come together, and then provide a brief case study of a project that evolved in the Australian Aboriginal and Torres Strait Islander primary healthcare context, which has both informed and been informed by the concepts reflected in this model. The model draws on a number of concepts that have been described in various ways in the literature, but that to our knowledge have not been drawn together in the way that is reflected in this paper.

### A systems-based partnership learning model

The model integrates key concepts and approaches to increase health system capacity to bring about improvements in quality of care and to impact on population level health outcomes within an environment of increasing resource constraints. It comprises a set of components that support reflection on mechanisms and interactions that can enable large-scale change and that can be applied in many different contexts. At the same time, each component incorporates within it a set of practices or mechanisms that enable the application of that component.

**Figure 1:**
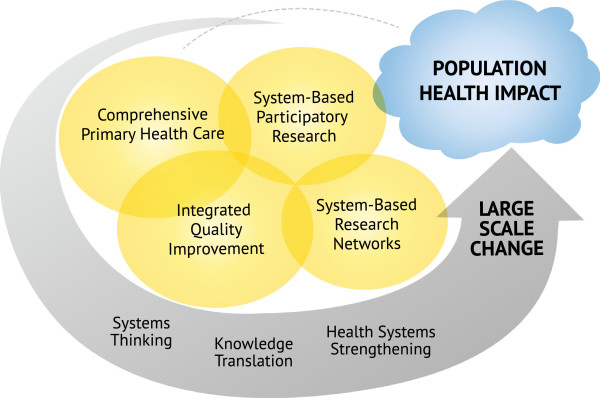
**Partnership learning model to achieve large-scale change.** The Partnership Learning Model integrates key concepts and approaches to increase health system capacity to bring about improvements in quality of care and to impact on population health. The components support reflection on mechanisms and interactions that enable health system strengthening and large-scale change. Within the Partnership Learning Model, Comprehensive Primary Healthcare is an approach to and operational base for health service delivery, Integrated Quality Improvement and Systems-Based Participatory Action Research are processes that generate and use data for the purpose of learning and improvement across the health system, and System-Based Research Networks provide a structure for multisite/multilevel learning and a mechanism for achieving influence at various levels of practice, management and policy—thereby enhancing potential for large scale change. Three approaches to thinking and using information that run through the model are systems thinking, health systems strengthening, and knowledge translation. Definitions and contributions of the concepts are summarized in Table 1.

In application, the model provides mechanisms to build or strengthen the capacity of a health system to continually work towards improving its performance through the establishment of partnerships and the development of resources and processes that enable healthcare teams to gather, analyse and use data for improvement. The PLM (Figure 1) illustrates how large-scale change can lead to improved population health outcomes through the interaction of four components: comprehensive primary healthcare (CPHC); integrated quality improvement (IQI); system-based research networks (SBRN); and system-based participatory action research (SBPAR).

Within the model, CPHC is an approach to and operational base for health service delivery, IQI and SBPAR are processes that generate and use data for the purpose of learning and improvement across the health system, and system-based research networks provide a structure for multisite/multilevel learning and a mechanism for achieving influence at various levels of practice, management, and policy. The definition of these components, and how their combined effects make the whole model greater than the sum of the components are summarized in Table 1 and discussed further below.

**Table 1:** Core definitions and contributions of components of the partnership learning model

**Core definition of key concepts as applied in the partnership learning model**	**Contribution of each component to the overall partnership learning model**
**Comprehensive primary healthcare:** An approach to PHC that aims to achieve equity in access to healthcare and other resources essential to health; reduced exposure to risk through changes in environmental and social determinants of health; improved participatory mechanisms, opportunities and political capabilities of marginalized population groups; increased inter-sectoral policy actions on the determinants of health; improved population health outcomes and greater health equity [20].	**CPHC** forms the operational base for the PLM through its focus on the needs and involvement of local communities, its inclusion of both patient-centered and population health approaches, and the recognition by the CPHC approach of the need to address the social determinants of health through co-ordinated, cross-sectoral action [21].
**Integrated quality improvement:** An interdisciplinary process designed to raise the standards of the care in order to maintain, restore and improve health outcomes of individuals and populations, and that includes the following key features:	**SBRNs** can draw on the combined effort of many participants from many levels of a health system, leveraging efficiencies of effort to meet priority challenges in a way that the usually more fragmented PHC sector can rarely achieve, and creating opportunities for large-scale ‘sense-making’ [42] and large scale change.
-The process is integrated into the core business of local organizations and the health system	**IQI and SBRNs** provide critical infrastructure, a systems focus, and an emphasis on generating and using data for ongoing improvement purposes. An **IQI** approach applied systematically but with sufficient flexibility to meet the needs of diverse stakeholders and contexts can provide a valuable source of data about the performance and state of system development of not only individual PHC services, but also collectively of services within a region or of the sector as a whole.
-Front line health workers, clinical leaders, managers and policy makers are engaged in QI processes	
-QI processes and tools are used to address multiple enablers of good quality care	
-Data on different enablers of performance are used to understand and inform system performance.	
**System-based research networks:** Research Networks that include multiple practices or services, managers, policy makers and others at various levels of the system in collaborative research to enhance the potential to understand and overcome system barriers to achieving better quality of care and health outcomes.	**SBPAR** supports ongoing, adaptive strengthening of health systems and the application and refinement of QI tools and processes in a way that meets the diverse needs of a variety of PHC services and the needs of key stakeholders across various levels and sectors of the system. This approach mobilizes a force for change and improvement and encourages better measurement and evidence to inform ongoing improvement efforts.
**System-based participatory research:** an approach that includes clinicians, office support staff, representatives of health related organizations, managers, and policy makers as well as community members in guiding the research process so that studies more closely match the needs of all stakeholders through their engagement; development of research design, methods and protocols so that the study is more amenable to participants and fits well with the local context; recruitment of research participants; data collection and analysis; and translation of results from the study back into the community, clinical practice, management and policy making.	The **SBPAR** approach has a strong **knowledge translation** orientation, and has relevance to the broader system strengthening required to support local PHC functioning, the need for regional managers and policy makers to understand diversity of services and factors influencing performance, and the need for local service staff to contribute to understanding how systems function and how they may be improved. The SBPAR approach encourages effective flow of tacit and explicit knowledge in multiple directions between a range of stakeholders from a range of different levels of the health system. The PLM can be seen as an integrated model for knowledge translation, and as such, provides an ongoing mechanism for strengthening health systems with the aim of delivering large-scale health benefits.
**Knowledge translation:** refers to the effective use of two types of knowledge within and across a range of levels within the health system. **Explicit knowledge** refers to codified knowledge, such as that found in research papers, systematic reviews and best-practice guidelines. **Tacit knowledge** refers to non-codified and experience-based knowledge [11-15]. Knowledge translation refers to active engagement by researchers with policy and practice issues (as experienced by policy makers and practitioners) and with research information, and application of that information to real challenges by people with deep understanding of the challenges and the context within which the information needs to be applied.	Effective **partnerships** are essential to each of the core components of the model and to achieving synergies between these components. Partnerships are important to effective engagement between stakeholders at multiple levels of the system, between stakeholders within different levels of the system, and across jurisdictional boundaries.
**Health systems strengthening:** the process of building the overall capacity of a health system, by ensuring the ‘six building blocks’ of a health system - service delivery; health workforce; information; medical products, vaccines and technologies; financing; and leadership and governance - are strong and integrated [1].	**Systems thinking** enables synergies between key components of the PLM.
**Systems thinking:** the in-depth consideration of the linkages, relationships, interactions and behaviours among the elements that comprise a complex adaptive system – i.e. one that self-organizes, adapts and evolves with time [2].	

There are three approaches to thinking and using information that run through the conceptual components of the model. Systems thinking involves in-depth consideration of the linkages, relationships, interactions and behaviours among the elements that comprise a complex adaptive system—*i.e.*, one that self-organizes, adapts, and evolves with time [2]. Health systems strengthening is the process of building the overall capacity of a health system, by ensuring the ‘six building blocks’ of a health system—service delivery; health workforce; information; medical products, vaccines and technologies; financing; and leadership and governance—are strong and integrated [1]. For research to contribute to health system strengthening, it should be focussed on important challenges for health system development, and the research findings need to be translated into practical applications—these are the domains of research translation, translational research, and knowledge translation [9,10]. Knowledge translation, for the purposes of this model, refers to the effective use of both research evidence and tacit knowledge [11-15] within and across a range of levels within the health system, and is consistent with the recently described concept of ‘integrated knowledge translation’ [16].

The PLM brings together these conceptual approaches and ways of thinking and using information to achieve the following objectives:

1. To support the implementation of best practice guidelines across various aspects of CPHC (clinical, health promotion, social determinants) in a way that fits with local circumstances.

2. To re-orient health systems (health center systems, regional support systems, and/or supportive policy environments) in line with best research evidence and local knowledge.

3. To enhance the capacity of information systems and healthcare teams to generate and use information effectively to support planning and evaluation at multiple levels of the system. This third objective, in particular, means the model is self-re-enforcing.

### Comprehensive primary healthcare

Primary healthcare is uniquely placed to address co-morbidity and to reduce health inequity [17]. Efforts to enhance the effectiveness of primary healthcare need to address both the systemic and clinical features of primary care [17,18]. Systemically, this requires equity of access and comprehensive healthcare coverage; clinically, it demands a focus on assessing and meeting the needs of individual people, families, and populations rather than on diseases [17]. A key aspect of systemic reforms is improved integration of PHC services with the broader health system, and enhancing the synergies between the health system building blocks [19] in the PHC sector through the use of systems thinking [2].

There are a number of features of primary healthcare services that present barriers to large scale QI programs, including:

Dispersion, diversity, and independence: the dispersed range of semi-independent local healthcare providers, working in a diversity of contexts, providing highly varied individual episodes of care, and operating in a loosely organized ‘system’ with varying levels of cooperation and coordination;

Scope and complexity of PHC: the broad scope of work of PHC, the requirement to provide holistic and patient centered care for people of all ages, for physical, psychological, and social conditions, and for diseases affecting any body organ;

Infrastructure to support a population health approach in PHC: the varied but generally limited state of development of ‘meso-level’ organizations supporting a population health approach in PHC; and

Information system capability: the generally poor and fragmented state of primary healthcare information systems and consequent lack of consistent and broad scale data on relative need, priorities, performance, and quality of care (partly as a result of the above challenges) [6].

The objectives of CPHC include equity in access to healthcare and other resources essential to health; reduced exposure to risk through changes in environmental and social determinants of health; improved participatory mechanisms, opportunities, and political capabilities of marginalized population groups; increased inter-sectoral policy actions on the determinants of health; and improved population health outcomes and greater health equity [20]. As such, CPHC forms the operational base for this PLM because of its focus on the needs and involvement of local communities, its inclusion of both patient-centered and population health approaches, and the recognition by the CPHC approach of the need to address the social determinants of health through co-ordinated, cross-sectoral action [21]. It provides an integrated approach to primary healthcare, incorporating preventive care, acute care, chronic illness care, population health, and health promotion, with attention to social appropriateness and accessibility, community engagement, community linkages, and advocacy.

A key challenge is how to most effectively enhance the potential of PHC to achieve its systemic and clinical objectives. Quality improvement concepts are increasingly being applied to this challenge with recognition that quality improvement approaches need to be applied at local, regional, and national levels to effectively strengthen health systems [22].

### Integrated quality improvement

The term ‘quality improvement’ has been used to describe one of four components of quality management (along with quality planning, quality control, and quality assurance) [6]. In a health context, it has been defined as ‘an interdisciplinary process designed to raise the standards of the delivery of preventive, diagnostic, therapeutic, and rehabilitative measures in order to maintain, restore, and improve health outcomes of individuals and populations’ [23]. There are a number of widely recognized quality improvement models, adapted from industry, including Total Quality Management (TQM), Continuous Quality Improvement (CQI), Business Process Re-engineering (BPR), Rapid Cycle Change (PDSA) models, Lean Thinking, and Six Sigma. Due to variation in implementation approaches and the varying contexts in which they are applied, there is limited evidence demonstrating the effectiveness of these models within the healthcare sector [6]. For example, there is positive but limited evidence on the effectiveness of QI collaboratives (that utilize a Rapid Cycle Change model) within the PHC sector. QI collaboratives bring together multi-professional experts from multiple organizations to work through structured activities to improve care in a specified area. Reviews of this approach have concluded that the effects are variable and there is a need for better understanding of factors that influence success [24,25]. Limitations of this approach within the broad responsibilities of CPHC include the focus on specific relatively narrowly defined topics (such as blood pressure, blood glucose control, or ‘access’) and that topics are usually determined ‘externally’ rather than by the local primary healthcare team.

While evidence indicates no single model of QI outperforms others, the most successful applications of QI in health systems are multi-site, multi-faceted approaches that aim to achieve change at various levels of the system [6,26]. The term ‘integrated’ is often used to describe such approaches. However, this term does not appear to have been clearly defined in relation to quality improvement models. Models that have been described as integrated [6,26] have been identified as having a number of key features that underpin success, including:

1. QI programs are integrated into the core business of the organization, rather than an add-on or one-off project.

2. Front line health workers, clinical leaders, and managers are engaged in QI processes.

3. QI processes and tools are used to address multiple enablers of good quality care (where ‘enablers’ cover structural, technological, political, cultural, educational, and emotional elements of an organization and its workforce).

4. Data on different enablers of performance—as identified above—are used to understand and inform broader system level performance, including through engagement of a variety of stakeholders at various levels of relevant organizations and through building networks across organizations.

We use the term integrated to reflect the incorporation in the PLM of the above four key features. With regard to IQI, the PLM has a systems focus on addressing quality of care issues, supporting implementation of evidence-based guidelines into routine practice and continually measuring clinical and system performance. Applying such IQI tools and processes to achieve large-scale health system strengthening is not unproblematic. A major challenge in the PHC context is the development and use of data systems that are adequately standardized to allow comparison over time and between health service units; sufficiently broad in scope to cover the priority areas of CPHC practice; and adaptable enough to meet the needs and organizational capacity of a diverse range of CPHC services and stakeholders. The development and implementation of effective QI systems therefore requires investment in appropriate QI tools, processes, and information systems; strengthening and re-orientation of service delivery systems to secure the potential to benefit from QI; and PHC clinical information systems that ensure the availability of robust and timely service population data. Another important part of the QI implementation infrastructure is the use of dedicated QI staff to build knowledge and skills and facilitate collaborative processes and action. As with other processes of implementing change in health services [27], the use of QI facilitators has been shown to enhance engagement of staff and the uptake of QI processes in PHC [28,29].

Both the application of QI methods and research into their effectiveness requires a systems focus [30]. Network structures are one approach to effect change at the broader system, organizational, and cultural levels [31].

### System-based research networks

In efforts to improve the linkage of research production to implementation, the 21^st^ century has seen rapid growth in Practice-Based Research Networks (PBRNs) in primary healthcare, particularly in the United States [32,33]. PBRNs link multiple dispersed practices or services in collaborative research, and have been promoted as a powerful mechanism to enhance the knowledge base of primary healthcare [34]. The networks draw on the experience and insight of local front line teams in framing research questions relevant to the PHC context, catalyzing local knowledge with academic expertise, and thus creating opportunities to apply rigorous research methods to important questions within the primary care setting. Successful PBRNs have ‘recognized that for researchers and clinicians to choose to work together for an extended period of time, they must focus on outcomes that are relevant to clinical practice, that is, solutions to the challenges that clinicians and their patients face on a frequent basis’ [23]. PBRNs facilitate the development of learning environments, provide vital infrastructure for knowledge translation in primary healthcare, and bridge the gaps between research and quality improvement and between researchers and practitioners [23].

While not all PBRNs incorporate QI approaches, PBRNs provide a potentially useful infrastructure to support standard use of QI tools and data, and to learn how other organizations address priority aspects of CPHC. These tools and processes can then be used in a way that suits local purposes and provides locally relevant and timely data, and to examine trends in key indicators over time. They allow PHC services to benchmark against comparative data from other PHC services in their district, region, or national level [23]. This has the potential to create a powerful data resource for management and research purposes.

Extending the concept of PBRNs to SBRNs involves including policy makers and managers in the knowledge translation process, thus enhancing the potential to understand and overcome system/infrastructure barriers to the provision of high-quality care. Much evidence-based practice can only be implemented with appropriate policy and management support [23]. Further, the interaction of participants across professional, sectoral, and even jurisdictional boundaries amplifies the impact of a SBRN from a community of peers to a network where learning can occur in the spaces between disciplines or practices [35].

The extension of the PBRN concept to a SBRN concept creates the potential to include stakeholders that have an interest in and responsibility for priority social determinants of health, at local, regional, or national levels. SBRNs thus enable research and practice to achieve the comprehensive primary healthcare envisioned in the Alma Ata declaration [1], in addition to issues of clinical care. They enhance the potential to apply systems thinking to strengthening comprehensive primary healthcare systems through shared understanding of issues that are common across services, sectors, and jurisdictions, as well as understanding of diversity.

A robust SBRN also provides a mechanism with the potential to respond to the constantly changing and evolving requirements of improving complex adaptive health systems. SBRNs are well suited to facilitate understanding of, and capacity to influence the evolution of complex adaptive health systems.

### System-based participatory action research

There is growing recognition of the need for community involvement in PBRNs to increase relevance of research to real world settings [36,37]. A combined community-based participatory action research (CBPAR)/PBRN approach has the potential to serve a range of purposes: guiding the research process so that studies more closely match the needs of all stakeholders (including providers, patients, and community members); development of research design, methods, and protocols so that the study is more amenable to participants and fits well with the local context; facilitation of recruitment of research participants; enriching data collection and analysis; and allowing rapid translation of results from the study back into clinical practice and the community [37]. Application of CBPAR to PBRNs may mean the concept of ‘community’ is defined to include networks of clinicians, office staff, the people who use the services, as well as various local health related organizations (or combinations of these groups) [23,38].

The principles and practices of CBPAR provide a disciplinary basis for working within a community or SBRN. There are challenges in this approach - gaining the trust and respect of the community; ensuring equally shared power and control over research processes; and overcoming conflicts associated with priorities, values and beliefs [39]. The converse of these challenges are enablers of CBPAR [39]. These enablers have much in common with the identified success characteristics of PBRNs, such as partners having a shared interest and vision with key decisions made at the outset; established information sharing protocols; and high levels of trust, reciprocity and respect [40,41].

The PLM incorporates system-based participatory action research (SBPAR), an extended concept of CBPAR, through inclusion of stakeholders from multiple levels of the system in a learning and developmental process that aims to transform healthcare systems and improve practice through engagement in QI research [38]. This approach has a strong knowledge translation orientation, is consistent with the principles of IQI, and has relevance to the broader system strengthening required to support local PHC functioning, the need for regional managers and policy makers to understand diversity of services and factors influencing performance, and the need for local service staff to contribute to understanding how systems function and how they may be improved.

### The whole is greater than the sum of the components

A systems approach requires consideration of the synergies and ‘spaces between’ components of a system [2]. While the relative emphasis may differ, there are clear synergies between the principles, rationales, and benefits of the key components of the PLM—these synergies can be maximized through the application of systems thinking to the PLM. In summary, the key contributions of the different components to the overall model are as follows: IQI and SBRNs provide critical infrastructure for continuous learning, improvement, and the measurement of performance; SBRNs and SBPAR provide mechanisms and practices to achieve the cross-sectoral engagement and patient-centered focus necessary for CPHC; and SBRNs can draw on the combined effort of many participants from many levels of a health system, leveraging efficiencies of effort to meet priority challenges in a way that the usually more fragmented PHC sector can rarely achieve, and creating opportunities for large-scale ‘sense-making’ [42]. IQI, SBPAR, and SBRNs support ongoing, adaptive strengthening of health systems and the application of QI tools and processes in a way that meets the diverse needs of a variety of PHC services and the needs of key stakeholders across various levels of the system. This has the potential to create a powerful force for change and improvement and to address the need for better measurement and evidence to inform ongoing improvement efforts. All four components emphasize or enable practices that enhance equity and engagement.

A number of the synergies between model components warrant further discussion. First is the powerful and dynamic nature of learning that may occur in a PLM. It enables the application and interaction of two types of knowledge: explicit knowledge and tacit knowledge [11-15]. Explicit knowledge refers to codified knowledge, such as that found in research papers, systematic reviews and best-practice guidelines. Tacit knowledge refers to non-codified and often experience-based knowledge. Tacit knowledge is critical to the appropriate adaptation and effective implementation of interventions, programs and policies at the local level. The PLM described here relies on effective flow of tacit and explicit knowledge in multiple directions between a range of stakeholders from a range of different levels of the health system. Wenger [35] emphasizes the profound learning that may occur across the boundaries of professional communities of practice, ‘areas of unusual learning, places where perspectives meet and new possibilities arise.’ Importantly, the model enables the effective translation of knowledge and information by various partners, working within their own organizational/stakeholder networks and across networks, for application in the local and broader context in a way that contributes to learning and improvement in research focus, design, methods and tools, and in service design, practice, systems, and policies. The PLM can be seen as an integrated model for knowledge translation, where translation refers to active engagement by researchers with policy and practice issues (as experienced by policy makers and practitioners) and with research information, and application of that information to real challenges by people with deep understanding of the challenges and the context within which the information needs to be applied.

Second, underpinning the adaptive capabilities of social learning within this model are the existence and use of effective data systems and tools. An IQI approach applied systematically but with sufficient flexibility to meet the needs of diverse stakeholders and contexts can provide a valuable source of data about the performance of not only individual PHC services, but also collectively of services within a region or of the sector as a whole. Researchers have an important role in development of data systems and tools. However, the role of research within the model goes beyond methods and tools to scientifically address the challenges confronting health services and systems. The research enterprise can also create a relatively safe environment for robust and honest discussion of those challenges and how they may be addressed. SBRNs convened by research groups can provide a relatively neutral arena for stakeholders from different parts of the health system, whose interactions may often otherwise be characterized by competition for resources or power. Managing these perceptions by demonstrating integrity and building trust is critical to the successful facilitation of a SBRN.

Third, the establishment and maintenance of effective partnerships is perhaps the single most important requirement for the effective operation of the PLM. The full operation of the model relies on effective partnerships between stakeholders at multiple levels of the system, between stakeholders within different levels of the system, and across jurisdictional boundaries. These partnerships are dependent on stakeholders seeing the potential for the partnerships to help them achieve their organizational and/or personal objectives; recognition of the value of a systems approach, of the value of data, the value of research; an interest and commitment to shared learning and in the potential for improvement across the system; and recognition and respect for the roles and challenges faced by different partners. Effective partnerships are essential to each of the core components of the model and to achieving synergies between these components. Key sources on the evidence of what makes for effective partnerships identify a number of important points common to the literature on PAR, IQI, and PBRNs, including: structures that facilitate two-way flow of information (including information sharing protocols), partners having a shared interest and vision with identification of achievable goals, high level engagement and commitment by partners, effective and committed leadership at senior level, the importance of local champions/leaders, and high levels of trust, reciprocity, and respect. The literature on effective partnerships also highlights the importance of good accountability arrangements with systematic and regular monitoring, and clear and agreed lines of responsibility [40,41,43].

### Case study: audit and best practice for chronic disease (ABCD) project

This case study describes the evolution of a SBRN through a program of research aimed at enabling community-based PHC services to provide high quality care to Aboriginal and Torres Strait Islander populations/communities. The case study shows how the Audit and Best Practice for Chronic Disease (ABCD) Project has both informed, and been informed by, the concepts reflected in the PLM, and illustrates the synergies between the key components of the PLM. Table 2 sets out the key points of focus and outcomes for research and health systems strengthening, over various stages of the evolution of the project.

**Table 2:** Research and health system focus of ABCD program of work

	**ABCD**	**ABCD extension**	**One21seventy**	**ABCD national research partnership (2010 – 2014)**
	**(2002 – 2006)**	**(2005 – 2009)**	**(2010 – )**	
Research questions	Could a QI approach be feasible and effective in Indigenous PHC services?	What was required to support large-scale implementation of the ABCD model?	No direct research function. Voluntary contribution of data by services for research purposes, and potential for other involvement of services in research	Understanding variation in quality of care and strategies for improvement.
				Exploring feasibility/functioning of a national system-based research network.
Health system strengthening dimension	QI approach embraced as way of improving (and demonstrating) quality of care.	Informed health system planning and policy by showing how the ABCD approach could be scaled up, examined barriers/enablers to engagement and improvement.	Provides QI training and tools with systems thinking focus; web-based data reporting system able to produce local and aggregated data reports, with benchmarking.	Brings together stakeholders from across jurisdictions and levels of health system to support and guide research on priority CPHC health system issues, contribute to refinement of QI tools and processes, interpretation of data, application of findings, and share lessons.
	Systems assessment tool (SAT) provided a mechanism for ongoing local system improvement and integration with other organizations and sectors (CPHC focus)			
		130+ health services and staff exposed to and used ABCD QI tools and processes over the 5-year project.		
			200+ health services using ABCD tools and processes by early 2013.	
Research findings	QI approach was well accepted, demonstrated feasibility of application of tools and processes, and improvements in care and intermediate health outcomes	Identified key barriers and enablers to scaling up in Aboriginal and Torres Strait Islander context	Increasing numbers of services that are engaged with One21seventy are participating in the ABCD Research Partnership – now about 70% – demonstrating increasing trust and interest in research.	To date focus has been on development of the Partnership, defining priority research questions, development of protocols and implementation of research, development of new priority tools.
Health system strengthening outcomes	Improvements in quality of care delivered + some intermediate health outcomes	Three State/Territory governments and a number of regional health services elected to implement the ABCD approach on a broad scale. Increased interest and support for QI. ABCD tools made available for use in a national QI program and for broader use by individual services and health affiliates.	Three State/Territory health services and several regional and local health authorities contract One21seventy to provide QI support to over 200 PHC services in five Australian States/Territories, including commitment of infrastructure support for QI.	Regional and national priority research questions being addressed through collaborative research
	Improved morale and team-building			Inter- and intra-jurisdictional and regional relationships strengthened.
	Findings and data used by health leaders advocate for systematic QI processes.			Data and research findings used by health leaders for advocacy purposes to advance system strengthening.
Further questions raised	How could the approach be scaled up for more widespread application?	Highlighted large variations in quality of care across health services—how could this be understood, addressed?	How best can a not-for-profit operate sustainably to support health-system strengthening in the Aboriginal and Torres Strait Islander context?	What is the impact of a systems-based research network (SBRN), itself a complex adaptive system (CAS) operating on other CASs?
			What is the impact of the One21seventy QI support service?	What will enable the effective engagement of stakeholders with responsibility for social determinants of health in a CPHC-oriented SBRN?

### Setting

Aboriginal and Torres Strait Islander people experience poor outcomes across a range of socioeconomic indicators including housing, education, employment, and health status [44]. These outcomes translate into a disparity in life expectancy of approximately 10 to 12 years [45] between Aboriginal and Torres Strait Islander people and other Australians, which is largely attributable to non-communicable diseases [46].

### Challenge

Primary healthcare provision in Australia is loosely organized with varying responsibilities split across federal and state governments. Inappropriate service delivery models and lack of access have aggravated the health gap [47].

### Approach

#### Phase one: exploring feasibility and acceptability of QI tools and processes

The ABCD Project began in 2002 to investigate organizational systems for prevention, early detection and management of chronic disease in primary healthcare centers. Using participatory action research approaches, an adapted Plan, Do, Study, Act process was introduced to 12 health centers in the Northern Territory to assess if this QI methodology was effective and acceptable within this context. Evidence-based clinical audit and systems assessment tools were developed for use by health center staff in annual QI cycles. These tools and processes enabled health centers to identify and address significant barriers to service delivery, resulting in an increase in the percentage of overall guideline-scheduled services delivered and improvement in intermediate health outcomes over two cycles of assessment [48,49].

#### Phase two: exploring scalability and expansion of IQI

An extension phase of the research project investigated system requirements for large-scale uptake and implementation of the ABCD tools and approach into routine practice in Aboriginal and Torres Strait Islander primary healthcare settings. Regional hub coordinators were employed to facilitate implementation and new tools were developed and introduced to address other priority areas of PHC. This phase of the work enhanced understanding of context-specific explanations for effective QI implementation [5,28,50-52]. This extension phase showed large variation between regions and between individual health centers in delivery of guideline-scheduled services [50].

Highly engaged management committees with strong stakeholder representation contributed to development of system-based participatory action research and system-based research networks. Clinical, policy, and management champions were influential in spreading the story of the ABCD tools and approach within and across organizations and jurisdictions.

#### Phase three: supporting wide scale implementation of IQI and development of a Partnership Learning Model

A spin-off, not-for-profit organization, One21seventy [53], was established to continue the health service support role after the research project ended. One21seventy provides an IQI framework that includes audit tools for a range of major chronic diseases, mental health, maternal and child health, rheumatic heart disease, and health promotion. QI tools are under development for application in other identified priority areas (including community food supply, environmental health and housing, youth health, and sexual health) to support a comprehensive PHC approach. One21seventy provides education and training in QI and access to a web-based data reporting system that allows real-time analysis and reporting of local level audit results, benchmarked against regional and national performance.

The ABCD National Research Partnership was established in the third phase of the project to increase the research and learning capability of the ABCD program of work. The research focus of the ABCD Partnership project is on understanding and ameliorating variation in the quality of care across health services. The Partnership forms a SBRN that supports engagement of key stakeholders across all levels of the health system, linking end-users and policy-makers directly with the research, and thereby facilitating knowledge translation [54]. Six-monthly meetings of partners and other stakeholders from all regions provide a mechanism for regular sharing of knowledge, identification and clarification of research priorities, improvement of the partnership and the systems that support it, and reporting of progress in health system strengthening, clinical outcomes, and research.

### Summary of evolution of the Partnership Learning Model

This case study shows how the core components of the model have evolved progressively through the phases of the ABCD project. The development and use of the IQI and SBPAR concepts were particularly important to the first phase—feasibility and acceptability of QI tools and processes. The significance of these two concepts increased substantially over the second and subsequent stages of the project. While the concept of CPHC was relevant to the first phase of the project in terms of a general approach to PHC, this concept also became increasingly relevant in the second and subsequent phases as new tools were introduced to support improvement in other priority clinical areas, health promotion, and community engagement. The concept of SBRNs was of emerging relevance in the second phase of the work, and became of vital significance in the third phase of the work.

## Discussion

While quality improvement approaches provide potential for ongoing learning, alone they do not offer the potential to engage with complexity offered by the combined power of SBPAR and SBRNs. Overall, the PLM provides a model for establishing an infrastructure and process for continuous learning and translation of evidence into practice. The model enables the effective translation of knowledge and information by various partners, working within their own organizational/stakeholder networks and across networks, for application in the local and broader context in a way that contributes to learning, and improvement in research focus, design, methods, and tools, and in service design, practice, systems, and policies. The PLM can be seen as an integrated model for knowledge translation, and as such, provides an ongoing mechanism for strengthening health systems with the aim of delivering large-scale health benefits.

There is little indication in the literature that the key components that make up the PLM are being integrated and applied in other settings in the way described in this paper. Few models for knowledge translation (or ‘research translation’ or ‘translational research’) incorporate ongoing capacity for the application of evidence into practice or practice into evidence; instead, most appear to provide frameworks for the diffusion or dissemination of one-off innovations.

There are features of the Australian environment that have enabled the evolution and development of the model, including the alignment of the ABCD program objectives with the service sector and the broader policy environment, and the fit of the tools and processes with existing incentive and regulatory frameworks and service systems. Of particular relevance in Australia is the establishment of accreditation requirements for primary healthcare services, and recent inclusion of engagement in QI as a requirement of accreditation. International differences in the relative priority of quality of care issues in the development of primary healthcare systems, differences in regulatory and governance frameworks, and differences in resources may limit the application of the model in some settings. However, quality improvement approaches have been identified as one of few strategies to support implementation of cost-effective interventions for which there is evidence of effectiveness in low- and middle-income countries [3], and many of the features of primary healthcare services that are described early in this paper as presenting barriers to large-scale QI programs are common to primary healthcare systems around the world. The PLM concept may therefore assist large-scale improvement efforts in other settings. The value placed by service providers and health authorities on the QI framework in providing common tools and comparable data, and of the QI framework in providing a shared language, comparative measurements, and opportunities to discuss the relative value of different strategies for implementation [28] is likely to have international relevance. The findings of research on the importance of organizational commitment, leadership at all levels of the system, and dedicated resources for successful implementation of the ABCD tools and processes [28] is also likely to be relevant to implementation of this type of model more generally. The PLM provides infrastructure and collaborative processes to support, evaluate and refine such efforts in a way that can be shaped by local stakeholders to fit with the local context.

The availability of funding schemes to support research-service-policy partnerships in Australia has also been vital to the development of the model. While service and policy partner organizations have provided in-kind and some funding support, to date the ABCD work has been funded largely through research grants. Questions about how to achieve an appropriate balance between funding QI activity as part of routine service delivery and funding QI related research, are vital to developing and sustaining this type of ‘public-good’ model. While QI is increasingly been seen as a core health service activity, government support for QI—and particularly support for the type of model described in this paper—may be vulnerable to shifts in priorities and perceived responsibilities as State and Commonwealth government reform initiatives unfold. Research grant funding is less susceptible in the short term to changes in political priorities, but is always time limited and should be focused on research rather than service delivery. In QI, and in the PLM specifically, there is a clear role for research in contributing to refining and evaluating existing tools and processes, and in developing and trialling innovations. For example, areas for further development in the ABCD program include strengthening processes to support engagement with data for QI purposes at regional and national levels; and exploring potential to engage local community members in efforts to improve quality of care. Researchers have a key role in building a culture of enquiry and curiosity, and to support development of appropriate information systems for generation and analysis of data, to facilitate the appropriate interpretation of data, and to provide a source for reference and reflection on local evidence in relation to the broader evidence base. Development of effective CBPAR and PBRN processes is demanding of researchers time and effort, and may require an attitudinal shift for many researchers. Their role in this model is to contribute methodological expertise. They may have less influence over the goals of the research than other partners, and implementation of actions arising from the research findings is likely to take precedence over journal publication [23].

At least one recent meeting of international leaders in implementation science has identified the need to advance research, practice, and policy to accelerate the scale-up and spread of effective health programs [55]. In response to this need, the PLM shows some potential for achieving wide scale engagement of researchers, practitioners, managers, and policy makers in efforts to scale up and spread effective health programs. The ABCD case study provides an example of the practical application of the PLM. This paper has focused on describing and illustrating the model to provide a basis for separate papers that will describe the trends in service quality that can be demonstrated through the engagement of key partners with this sort of partnership. Important questions are now emerging about the sustainability of the PLM in Australia, and its potential for growth, adaption, or replication, both in Australia and internationally.

## Competing interests

RB is the Scientific Director of One21seventy, a not-for-profit entity within Menzies School of Health Research that provides QI support on a fee for service basis to primary healthcare services across Australia. None of the authors receive financial support from One21seventy, and One21seventy is not providing any financial support for the preparation of this manuscript. The authors have no other competing interests in the preparation of this manuscript.

## Authors’ contributions

RB conceived and had the primary role in drafting the manuscript; VM and JB played substantial roles in reviewing the literature and drafting the manuscript; GS provided intellectual input in the development and refinement of the manuscript. All authors read and approved the final manuscript.
